# Near Infrared Laser-Induced Content Release from Magnetic Bead-Encapsulated Giant Unilamellar Vesicles

**DOI:** 10.17912/micropub.biology.002027

**Published:** 2026-03-25

**Authors:** Hiromasa Shiraiwa, Koichiro Akiyama, Tomoyuki Kaneko

**Affiliations:** 1 Major in Frontier Bioscience, Graduate School of Science and Engineering, Hosei University, Tokyo, JP

## Abstract

The spatiotemporal control of content release from liposomes is crucial for the development of drug delivery systems (DDS). In this study, we established a system to induce content release from giant unilamellar vesicles (GUVs) encapsulating magnetic beads by irradiating them with a 1480 nm near-infrared laser. This approach utilizes local photothermal conversion to destabilize the membrane physically. The resulting content release process was directly visualized using fluorescence microscopy, demonstrating the utility of this method for analyzing the dynamics of membrane deformation and fissure formation.

**Figure 1. NIR laser-induced release of magnetic beads from GUVs:**
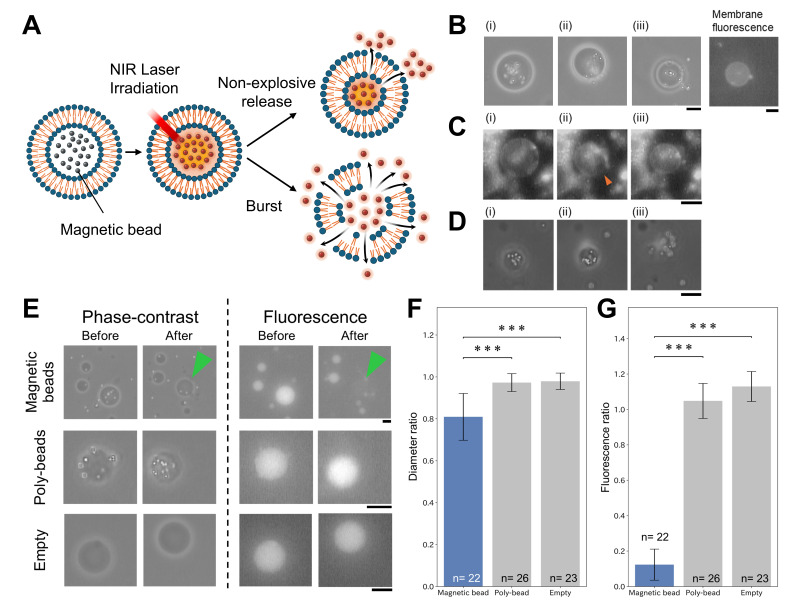
(A) Schematic illustration of NIR laser irradiation on magnetic bead-encapsulating GUVs, showing both non-explosive and burst modes. (B) Phase-contrast time-lapse images of the non-explosive release mode. (i) Immediately prior to irradiation. (ii) At 24.8 s after the irradiation onset, the magnetic beads were released toward the bottom right. (iii) 4.4 s after (ii), where the released magnetic beads spread into the external aqueous phase. The corresponding membrane fluorescence image of (iii) shows no fluorescence in the region of the released magnetic beads. (C) Time-lapse fluorescence images capturing the moment of transient fissure formation. (i) During NIR laser irradiation, the GUV maintained a stable spherical shape prior to fissure formation. (ii) At 66 ms after (i), a "transient fissure" is formed in the direction indicated by the orange arrowhead. The fissure was resealed within 66 ms. (iii) The GUV returned to a completely spherical shape following resealing. (D) Phase-contrast time-lapse images showing vesicle bursting. (i) Pre-irradiation. (ii) During irradiation. (iii) Burst event 9.2 s after irradiation onset, showing that the magnetic beads were scattered around the GUV center. (E) Phase-contrast and fluorescence images of empty, polystyrene bead-encapsulated, and magnetic bead-encapsulated GUVs before and after NIR laser irradiation. Calcein was used to visualize the internal aqueous phase. In the magnetic bead-encapsulated GUV, the green arrowhead indicates the position of the liposome after irradiation, where internal fluorescence was no longer detectable. Scale bars in (B–E) represent 10 µm. (F)&nbsp;Diameter ratios (D _post_ /D _pre_ ) before and after irradiation under the three GUV conditions. The ratio significantly decreased only in the magnetic bead-encapsulated GUVs, indicating a reduction in the GUV diameter due to NIR laser irradiation. Error bars represent standard deviation (***p < 0.001 by Student’s t-test).(G) &nbsp;Fluorescence intensity ratios (F _post_ /F _pre_ ) before and after irradiation for the three GUV conditions: magnetic bead-encapsulated, polystyrene bead-encapsulated, and empty GUVs. This ratio significantly decreased only in the magnetic bead encapsulated GUVs, indicating a loss of internal fluorescence. Error bars represent standard deviation (***p < 0.001 by Student’s t-test).

## Description

Drug delivery systems (DDS) using giant unilamellar vesicles (GUVs) are promising technologies for controlling pharmacokinetics (Allen & Cullis, 2013). Maximizing therapeutic efficacy requires stable drug retention during transport and rapid release upon reaching the target site of action (Mura et al., 2013). However, passive diffusion or environment-dependent mechanisms (e.g., pH or enzymes) lack precise spatiotemporal control, posing a risk of off-target effects. To overcome this, "on-demand" DDS triggered by external stimuli are being developed. Near-infrared (NIR) lasers are particularly useful as triggers because of their deep tissue penetration and minimal invasiveness (Sershen et al., 2000). Photo-responsive carriers using gold nanomaterials (Wu et al., 2008 ; Schramm et al.,2025) or chemically modified lipids (Carter et al., 2014) have been widely reported. However, incorporating these nanomaterials into the lipid bilayer often requires complex chemical modifications and intricate molecular design. While magnetic nanoparticles (Fe _3_ O _4_ ) are typically employed for drug release triggered by alternating magnetic fields (AMF) via thermal (Fortes Brollo et al., 2020) or mechanical mechanisms (Vlasova et al., 2019), they are also known to exhibit efficient photothermal conversion under NIR irradiation, a property utilized in hyperthermia cancer therapy (Chu et al., 2013). Here, we constructed a novel release system using magnetic bead-encapsulating GUVs, leveraging the localized photothermal effect of NIR irradiation. This system induces cargo release via physical membrane destabilization without the need for chemical modification of lipid components, providing a foundation for next-generation optically controlled DDS.

GUVs were prepared from DOPC (base lipid) and doped with Atto488-DOPE (1:1,000 mole ratio) or Rhod-PE (1:100 mole ratio). These GUVs encapsulated either magnetic (Fe _3_ O _4_ ) microbeads or polystyrene beads. All observations were performed on an inverted light microscope (Olympus IX71) equipped with a CCD camera operating at 30 fps, using phase-contrast microscopy and epifluorescence microscopy. Phase-contrast images were used to track bead motion, while fluorescence images were used to monitor calcein retention (aqueous-phase leakage) and membrane fluorescence. Internal fluorescence intensity was quantified from the recorded images using ImageJ to evaluate leakage of the encapsulated aqueous phase.

When the magnetic bead-encapsulated GUVs were irradiated with an NIR laser, the release of the magnetic beads was confirmed. Two distinct release patterns were identified, as summarized in Figure 1A . These consist of a non-explosive release mode, in which beads were released while the GUV retained its vesicular structure, and a burst mode, in which the GUV ruptured explosively, releasing all its contents into the outer phase.

Irradiation with a 1480 nm NIR laser primarily induced a non-explosive release phenomenon ( Figure 1B ). In this mode, the GUV maintained its overall shape during bead release. Fluorescence imaging confirmed that the released beads were free of membrane lipids, indicating selective cargo release. As shown in the video, continuous observation of membrane fluorescence revealed the formation of a transient slit-like opening in the membrane during irradiation ( Figure 1C, Extended data). We defined this opening as a "Transient Fissure." This membrane deformation lasted approximately 67 ms, during which the beads were discharged from pores. Subsequently, the fissure closed instantaneously, and the GUV recovered its spherical shape with a reduced diameter. In contrast, burst mode, characterized by complete vesicle rupture, occurred in only 15.9% of the observed events ( Figure 1D ).

To verify the release specificity, we compared magnetic bead-encapsulating GUVs with two control groups: GUVs encapsulating polystyrene beads and empty GUVs ( Figure 1E ). All types of GUVs encapsulated the water-soluble fluorescent dye, calcein, to monitor the retention of the inner phase. Upon irradiation, magnetic bead-encapsulating GUVs released the beads, and post-irradiation imaging confirmed a significant loss of internal calcein fluorescence, indicating leakage of the inner solution. In contrast, polystyrene bead-encapsulating GUVs showed neither bead release nor leakage of calcein fluorescence. Visually, while magnetic bead-encapsulated GUVs shrank after irradiation, the diameters of polystyrene bead-encapsulated GUVs remained unchanged. Similarly, empty GUVs showed no decrease in calcein fluorescence following irradiation.

We first quantified the morphological changes ( Figure 1F ). The diameter ratio was defined as Dpost/Dpre. The diameter ratio of the magnetic bead-encapsulating GUVs was 0.81 ± 0.11 (mean ± S.D., n = 22), indicating a significant size reduction concomitant with leakage. In contrast, polystyrene bead-encapsulating GUVs maintained their size, with a ratio of 0.97 ±0.04 (n = 26). The empty GUVs exhibited stability similar to that of the polystyrene controls.

Next, we quantified the change in the internal content ( Figure 1G ), including empty GUVs (containing no beads) as an additional control for the experiment. The fluorescence ratio was defined as Fpost/Fpre (where F represents the background-subtracted internal fluorescence intensity before and after irradiation). The fluorescence ratio for magnetic bead-encapsulating GUVs was 0.12 ± 0.09 (n = 22), showing a decrease to 12% of the initial intensity. Conversely, the ratio of polystyrene bead-encapsulating GUVs remained high at 1.04 ± 0.09 (n = 26). The empty GUVs also exhibited no fluorescence loss.

In this study, we demonstrated that NIR laser irradiation of liposomes encapsulating magnetic beads, which act as photothermal conversion agents, induces pore formation and subsequent cargo release. Control experiments using polystyrene beads suggest that the observed transient fissures are primarily driven by thermal destabilization of the membrane, rather than mechanical bead movement associated with laser-induced fluid convection. While direct, simultaneous observation of bead translocation through these transient fissures remains challenging due to optical interference from the NIR laser during phase-contrast imaging, the synchronized occurrence of membrane fissure formation, leakage of the internal aqueous phase (loss of calcein), and the subsequent reduction in GUV diameter (Figures 1F and 1G) strongly suggests that these transient pores serve as the primary exit route for the encapsulated cargo. In addition, a technical challenge was noted: the convective flow generated by the NIR laser frequently caused the GUVs to migrate, preventing continuous irradiation in several samples. To address this, the future implementation of GUV trapping mechanisms using microfabrication techniques, such as photolithography for physical barriers or electric-field-based dielectrophoresis, would significantly facilitate the stable, real-time observation of the release behavior (Korlach, J., et al., 2005).

Furthermore, two distinct release modes were observed: a transient fissure mode, in which the pore reseals, and a burst mode, characterized by irreversible membrane rupture. The specific mechanisms underlying the occurrence of each mode remain unclear. Future studies require precise control of laser power to determine the biophysical membrane factors that govern the transition between these release modes. Ultimately, this model contributes to the fundamental understanding of lipid membrane dynamics and cellular morphological changes under nonequilibrium conditions and serves as a potential experimental platform in membrane biophysics.

We established a model for cargo release from GUVs driven solely by physical energy, achieved by irradiating magnetic bead-encapsulated GUVs with an NIR laser. Notably, this approach eliminates the need for sophisticated phospholipid engineering and the use of specialized photothermal agents. This model serves as a valuable experimental platform for investigating lipid membrane dynamics and the physical principles underlying cellular morphological changes under nonequilibrium conditions of the cell membrane. Furthermore, such liposomal cargo release systems are expected to provide a foundation for next-generation light-controlled drug delivery systems (DDS).

## Methods

**GUV Preparation**

GUVs were prepared using a water-in-oil (W/O) emulsion transfer method. An inner phase (2 µL) containing 30% (v/v) Percoll, 200 mM sucrose, 1 mg/mL BSA, and magnetic beads (particle concentration: 3.6–6.1 × 10⁹ beads/mL) was added to 20 µL of the lipid solution (1 mM DOPC and 1 µM Atto 488-DOPE in MCT oil) in a 1.5 mL microtube. The mixture was emulsified by vigorously rubbing the tube on a metal tube rack. The emulsion was then layered onto 30 µL of the outer phase (100 mM glucose and 1 mg/mL BSA) in a PCR tube. To facilitate the passage of droplets through the lipid monolayer at the oil–water interface, the samples were centrifuged at 1,000 × g for 5 min at 25°C. For centrifugation, a custom adapter was used, consisting of a lidless 1.5 mL microtube stacked with a shortened 1,000 µL pipette tip.

After centrifugation, the upper oil phase was discarded. The bottom 10 µL containing the GUVs was collected and transferred to a new PCR tube containing 10 µL of fresh outer solution. The suspension was centrifuged again (1,000 × g, 1 min, 25°C) for washing. Finally, the bottom 10 µL was transferred to a fresh tube, and the GUVs were resuspended by gently tapping the tube.

For the experiments shown in Figure 1B, the concentration of Atto 488-DOPE in the lipid solution was adjusted to 10 µM. For Figure 1C, 10 µM Rhod-PE was used instead of 1 µM Atto 488-DOPE. For Figures 1E, F, and G, 1 mM calcein was added to the inner phase of the droplets. In the control experiments, polystyrene beads were included in the inner phase instead of magnetic beads.

**Observation Chamber**

Observation chambers were constructed on glass slides using two narrow strips of double-sided tape as spacers. A 4 µL aliquot of the GUV suspension was deposited between the strips using a micropipette. A coverslip was placed on top, and the chamber edges were sealed with VALAP (a mixture of beeswax [Wako, CAG5129], lanolin [Wako, CAK2960], and vaseline [Wako, SKL0870]) to prevent sample evaporation.

**NIR Laser Irradiation**

Individual GUVs were irradiated with a 1480 nm near-infrared (NIR) laser (IPG Laser GmbH, Germany) to observe morphological changes and bead-release behavior in real time. The irradiation position was fine-tuned using a motorized XY stage system (SC-101, Sigma Tech), and the laser onset/offset was controlled using a single-shutter controller (SSH-C1R, Sigma Koki). As controls, GUVs encapsulating polystyrene beads and empty GUVs were irradiated under the same conditions. The irradiation duration was defined as the time from laser onset to the first visible release of the bead. As shown in Figures 1F and 1G, the control groups were irradiated for a duration exceeding the average release time of the magnetic beads.

**Image Acquisition and Analysis**

The dynamic behaviors were recorded as videos at 30 frames per second (fps). All image analyses, including frame extraction, diameter measurement, and fluorescence intensity quantification, were performed using the Fiji software ( https://imagej.net/software/fiji/ ).

**Use of Artificial Intelligence Tools**

Gemini 3 Pro and Paperpal were used solely for language refinement and editorial improvement. Generative AI was not involved in drafting the original manuscript text, generating the scientific content, or interpreting the experimental results. All AI-assisted revisions were carefully reviewed and approved by the authors of this study.

## Reagents

**Table:** 

**Reagents**	**Full name**	**supplier**	**Catalog number**
Glucose	Glucose	FUJIFILM Wako Pure Chemical Corporation	049-31165
Sucrose	Sucrose	FUJIFILM Wako Pure Chemical Corporation	194-00025
Percoll	Percoll	GE Healthcare	17-0891-01
BSA	Bovine Serum Albumin	FUJIFILM Wako Pure Chemical Corporation	019 23293
Calcein	3,3'-Bis[N,N-bis(carboxymethyl)aminomethyl]fluorescein	FUJIFILM Wako Pure Chemical Corporation	340-00433
DOPC	1,2-Dioleoyl-sn-glycero-3-phosphocholine	Tokyo Chemical Industry	4235-95-4
Atto 488 -DOPE	1,2-Dioleoyl-sn-glycero-3-phosphoethanolamine labeled with Atto 488	ATTO-TEC	AD488-161
Rhod-PE	1,2-Dioleoyl-sn-glycero-3-phosphoethanolamine-N- (lissamine rhodamine B sulfonyl) ammonium salt	Avanti Polar Lipids	81010
MCT oil	Medium-Chain Triglyceride Oil	The Nisshin OilliO Group	4543925
magnetic beads	Dynabeads MyOne Streptavidin T1	Thermo Fisher Scientific	65601
polystyrene beads	Polybead Polystyrene Microspheres (2.5% Solids-Latex), 1.00μm	Polysciences	07310-15

## Data Availability

Description: Video of transient fissure formation, covering 1 s before and after the event (played back at 1/10 speed). The fissure appears at the 0.5 s mark. NIR laser irradiation had already commenced prior to the start of the video.. Resource Type: Audiovisual. DOI: https://doi.org/10.22002/1tkb4-g5h95
